# Efficacy of Mushroom Metabolites (*Pleurotus ostreatus*) as A Natural Product for the Suppression of Broomrape Growth (*Orobanche crenata* Forsk) in Faba Bean Plants

**DOI:** 10.3390/plants9101265

**Published:** 2020-09-25

**Authors:** Tamer Elsakhawy, Muneera D. F. ALKahtani, Ali A. H. Sharshar, Kotb A. Attia, Yaser M. Hafez, Khaled A. A. Abdelaal

**Affiliations:** 1 Microbiology Department, Soils, Water and Environment Research Institute (SWERI), Agriculture Research Center (ARC), Giza 12619, Egypt; drelsakhawyg@gmail.com; 2 Biology Department, College of Science, Princess Nourah bint Abdulrahman University, Riyadh POX 102275-11675, Saudi Arabia; mdfkahtani@gmail.com; 3 Weed Research Central Laboratory (WRCL), Agriculture Research Center (ARC), Giza 12619, Egypt; alysharshar1@gmail.com; 4 Center of Excellence in Biotechnology Research, King Saud University, Riyadh POX 2455-11451, Saudi Arabia; kattia1.c@ksu.edu.sa; 5 EPCRS Excellence Center, Plant Pathology and Biotechnology Laboratory, Agricultural Botany Department, Faculty of Agriculture, Kafrelsheikh University, 33516 Kafr El-Sheikh, Egypt; hafezyasser@gmail.com

**Keywords:** broomrape, faba bean, gas chromatography-mass spectrometry, chlorophyll concentration, anatomical characters, natural products, spent mushroom substrate extract, Roundup

## Abstract

Broomrape parasitism on faba bean (*Vicia faba* L.) is the most destructive factor for this crop in Egypt. Pot experiments were conducted during the two successive seasons 2017/2018 and 2018/2019 to study the mitigation of broomrape stress on faba bean using a ten-fold dilution of 10% (w/v) spent mushroom substrate extract (SMSE) of *Pleurotus ostreatus* and the same dilution of culture filtrate of mushroom (MCF) grown in potato dextrose broth (PDB) at a rate of 48 l hectare^−1^ compared with the commercial herbicide Roundup (Glyphosate 48% emulsifiable concentrate) at a rate of 144 cm^3^ ha^−1^ on the two varieties (Misr3 and Sakha3) cultivated in broomrape-infested soil. The treatments include the use of mushroom products as foliar spray and/or soil amendment in addition to Roundup spraying as a recommended treatment. Using Gas Chromatography-Mass Spectrometry (GC-MS) spectroscopy, our results indicate that the major components of the two mushroom products were bioactive compounds such as polyphenol and high molecular weight aliphatic and aromatic hydrocarbons that may interfere with parasite and host metabolism. These results indicated that SMSE of *P. ostreatus* and MCF of the same mushroom grown in potato dextrose broth (PDB) gave the best control of broomrape, and increased plant height, root length, leaf area, chlorophyll concentration, relative water content and seed yield (g plant^−1^), as well as anatomical characters of leaves in the two faba bean varieties (Misr3 and Sakha3), such as upper and lower epidermis, palisade tissue, spongy tissue and vascular bundles. Additionally, electrolyte leakage was decreased in the treated plants compared to control plants and the plants treated with Roundup (glyphosate) because of the important role of SMSE and MCF in the improvement of faba bean water status.

## 1. Introduction

Faba bean is one of the most important food crops worldwide, especially in Egypt, although the area of cultivation has shrunk in the last two decades from 178,531 hectares in 1991 to 32,532 hectares in 2017. There are many biotic and abiotic stress factors affect its growth and production, such as drought stress [1] and broomrape stress [2]. Broomrapes (*Orobanche* sp.) are a root hollow-parasitic plant lacking chlorophyll and exclusively depending on the specific host plant for nutrition [3]. They parasitize a wide range of plant species, including leguminous, solanaceous, oil plants, cruciferous in addition to medicinal plants [3]. They cause extensive yield losses in the host crops, especially in warmer and drier regions of Africa, Europe, and Asia, mainly in faba bean fields in Egypt [2,4,5]. Broomrape infestation led to decreased anatomical characters of faba bean leaf and stem, such as the thickness of lamina leaf, diameter of vascular bundles and thickness of phloem tissue in leaves. Anatomical characters of stems, such as stem diameter and vascular cylinder, were also decreased in infested faba bean plants compared with un-infested control plants. These negative effects of broomrape parasitization threaten the income of the farmers [3]. Broomrape seeds can be easily transported to other fields by means of agricultural tools, man, propagules such as crop seeds, and animals through ingestion and excretion of *Orobanche* seeds [3]. Since the primary stages of infection take place underground, injury to the host plant occurs before the emergence of the parasite shoots, which hinders the discovery of infection and the development of effective control strategies. In addition, a single *Orobanche* plant can produce more than 500,000 seeds, which can maintain its viability for about 20 years in the soil. This offers the parasite a wide diverse genetic pool which enables broomrape to withstand environmental changes and control strategies [6]. For all the previous reasons, the available control strategies against broomrapes have not introduced applicable, effective and economical solutions as predicted [7,8].

Broomrape control mainly depends on the use of resistant cultivars of the host plants and when they are not available, control depends on the avoidance of susceptible plant species in the crop rotation [9]. Glyphosate application reduced dodder weed (*Cuscta* spp.) and improved plant growth of infested Egyptian clover plants [10]. However, excessive use led to the emergence of herbicide-resistant weed, with Duke and Powles [11] reporting the negative effect of glyphosate-based herbicides on human and environment health. Glyphosate has been evaluated regularly by national and international agencies [12,13], and although glyphosate has relatively low toxicity in human and animals, the International Agency for Research on Cancer (IARC) reported that glyphosate and its based formulations are probably carcinogenic in humans [14,15,16]. The use of other chemical herbicides has shown limited success for the management of broomrape parasitism. This is because of the tight physical and metabolic relation between host plant and broomrape through the attachment to the host root, hence success in chemical treatment of attached parasites is restricted to few host-broomrape species pairs where herbicides are able to selectively hinder broomrapes without injuring the host crop [6,7,17]. Biological control may introduce an efficient alternative strategy in the broomrapes control under field conditions depending on the effectiveness of natural enemies as bioagents. 

The potential of myco-herbicidal microorganisms for parasitic weeds management have been studied [18]. The application of Mycorrhizal fungi combined with bacterial strains positively affected nodule numbers and total dry matter of faba bean plants under broomrape infestation [19]. Moreover, the application of bio-control agents such as *Trichoderma* spp. And *rhizobacteria* species improved plant growth characters and play pivotal role in controlling broomrape in faba bean plants [20]. 

Spent mushroom substrate extract (SMSE) is a legnocellulolytic substrate residue of edible mushroom cultivation [21]. It contains many bioactive compounds such as phenolic compounds, which can activate defense mechanisms of plants under biotic stress conditions. The application of SMSE led to activate the defense system of rice plants against *Pyricularia oryzae* infection, associated with phytoalexin accumulation and the expression of defense-related genes. Therefore, the aim of our research was to study the efficacy of SMSE containing the metabolites of mushroom grown under solid state fermentation on lignocellulitic biomass and MCF containing the metabolites of mushroom grown under submerged fermentation conditions as a natural product and mycoherbicidal agent against broomrapes parasitism and improve the growth and yield characters in stressed faba bean plants.

## 2. Results

### 2.1. GC–MS Analysis of the Ethyl acetate Extract of Both SMSE and MCF

Gas Chromatography–Mass Spectrometry (GC–MS) analysis of the ethyl acetate extract of both spent mushroom substrate extract (SMSE) and mushroom culture filtrate (MCF), shows that phenol, 2, 4-bis (1,1-dimethylethyl) was the most dominant compound in the SMSE where it represented approximately 58% of total measured compounds (Table 1), followed by 1,2-benzenedicarboxylic acid (phthalic acid), which represented about 11%, then 1-hexadecanol, 2-methyl-, which represented about 5% of total estimated compounds. Other compounds, such as thieno [3,4-c] pyridine, 1,3,4,7-tetraphenyl-, 1-hexadecanol, 2-methyl- (cetyl alcohol), 1-dodecanol, 3,7,11-trimethyl (lauryl alcohol) and 2-hexadecanol, represented about 4% for each (Table 1). Table 2 shows that the most dominant compound in the MCF was 2 (1h)-naphthalenone, octahydro-1-methyl-1-(2-p ropenyl)-, (1a,4Aa,8Aa) accounting for nearly 58% of total estimated compounds, followed by 1,2-benzenedicarboxylic acid with about 16% of the total compounds (Table 2).

Other compounds in the MCF existed with values ranging between 0.7–6%, like 2-Tridecanol, 1-hexadecanol and isochiapin B (Table 2). All compounds detected by GC–MS contain carbon atoms ranging between 14–31 in both extracts. The molecular weight of detected compounds ranged between 206–550 in case of SMSE and 200–610 in case of MCF. Compounds detected in both extracts included variable types of functional groups like phenols, acids, alcohols, esters and different types of carbon structures as aliphatic, aromatic and heteroaromatic structures (Table 1 and Table 2). 

### 2.2. Effect of SMSE, MCF and Roundup on Nutrient Elements and Morphological Characters of Faba Bean Plants Under Broomrape Infestation

In general, the vegetative parameters and nutrient elements analysis of faba bean plant at 60 days from sowing varied greatly with treatment and the nutritive potential of mushroom extracts represented by SMSE or MCF was clearly observed. The highest nitrogen, phosphorous and potassium (NPK) concentrations in plant tissue were recorded for the plants treated with MCF (M4, M5, M6, S4, S5, S6), followed by the plants treated with SMSE (M1, M2, M3, S1, S2, S3), in Table 3, while it was observed that plants treated with herbicide (M7, S7) recorded the lowest values after the control. Moreover, the treatments represented by spraying plus soil amendment (M2, M5, S2 and S5) of both extracts were the most effective, followed by spraying treatment (M1, M4, S1 and S4). 

The results obtained in Table 4 indicate that plant height, plant dry weight and root length were negatively affected with broomrape infestation. However, the application of MCF (M4, M5, M6, S4, S5, S6), followed by the plants treated with SMSE (M1, M2, M3, S1, S2, S3), improved the plant height, plant dry weight and root length. Additionally, SMSE and MCF of *P. ostreatus* led to a significant increase in plant height, plant dry weight and root length of faba bean plants under broomrape infestation compared to Roundup and control treatments (Table 4). The best results were recorded in the plants treated with 10% solution of spent mushroom extract + soil amendment with 10% MCF (M5), followed by the plants treated with soil amendment with 10% MCF (M6).

### 2.3. Effect of SMSE, MCF and Roundup on Phosiological and Yield Characters of Faba Bean Plants Under Broomrape Infestation

Table 5 showed that broomrape infestation significantly decreased leaf area (cm^2^), chlorophyll a and chlorophyll b concentrations of faba bean plants in both seasons. Conversely, the application of SMSE of *P. ostreatus* and MCF as well as Roundup led to a significant increase in leaf area (cm^2^), chlorophyll a and chlorophyll b concentrations. The best results were recorded with SMSE and MCF compared with other treatments in the tolerant cultivar. The best results were recorded in the plants treated with 10% solution of MCF + soil amendment with 10% MCF (M5) followed by the plants treated with soil amendment with 10% MCF (M6) in the tolerant cultivar. On the other hand, the best results of the previous studied characters in sensitive cultivar (Sakha3) were observed in the treatment M5 (Spraying + soil amendment with 10% MCF).

The data in Table 6 demonstrate that the broomrape number was higher in the sensitive cultivar (Sakha3). At this stage (60 days from sowing), broomrape would not appear on the soil surface, but could be counted as clusters attached to root vessels. The most potent treatment for reducing broomrape in the sensitive cultivar was the application of herbicide (S7), followed by the treatments where MCF was applied. In case of rhizobia nodules, the application of herbicide caused a decline in nodulation in both cultivars, while other treatments based on mushroom metabolites enhanced nodulation and it was reflected in both the nodule number and nodule dry weight in the two seasons.

The results presented in Table 7 show that plant dry weight, plant height and pod numbers of faba bean plants at harvesting stage were significantly decreased under broomrape infestation. However, using MCF caused a significant increase in plant dry weight, plant height and pod numbers of faba bean plants in the two seasons (M4, M5, M6, S4, S5, S6), followed by the plants treated with SMSE (M1, M2, M3, S1, S2, S3). Additionally, the maximum values of these characters were obtained with the M5 treatment (spraying + soil amendment with 10% MCF), followed by M6 treatment (soil amendment with 10% MCF) in the Misr3 cultivar. Moreover, the best results in sensitive cultivar (Sakha3) were shown in treatment S5 (Spraying + soil amendment with 10% MCF), followed by the S6 treatment (soil amendment with 10% MCF) compared with Roundup and other treatments in both seasons.

Infestation with broomrape led to a significant decrease in faba bean yield parameters at harvest (Table 8). Our results indicated that seed yield (g/plant), straw yield (g/plant) and 100-seed (g) significantly decreased in infested untreated faba bean plants. On the other hand, the application of SMSE and MCF caused a significant increase in seed yield (g/plant), straw yield (g/plant) and 100-seed (g) of faba bean plants in the two seasons. Furthermore, M5 treatment (Spraying + soil amendment with 10% MCF) gave the best results of the studied characters followed by M6 treatment (soil amendment with 10% MCF) in the tolerant cultivar (Misr3). Likewise, the best results in the sensitive cultivar (Sakha3) were shown in treatment S5, followed by S6 treatment, compared with Roundup and other treatments in both seasons.

**Figure 1:**
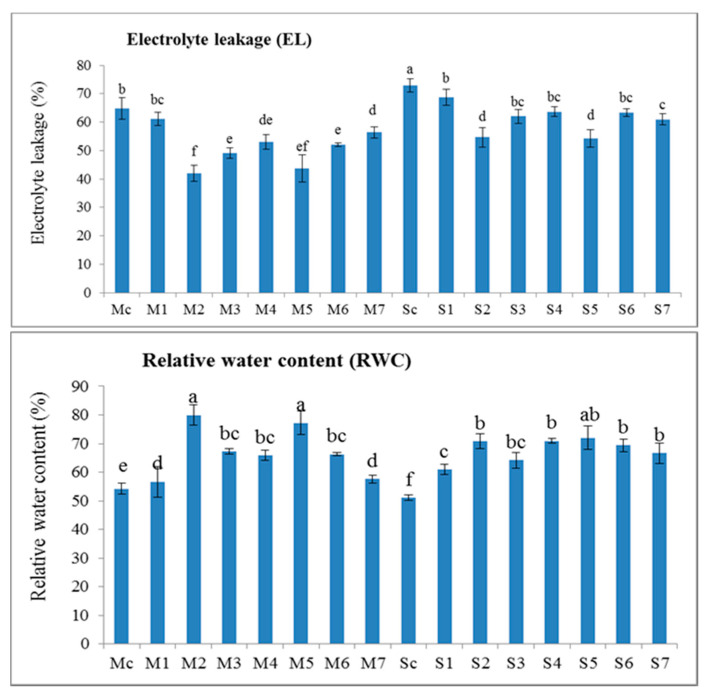
Electrolyte leakage and relative water content in leaves of the two varieties (Misr3 and Sakha3) faba bean plants under infestation with broomrape and treated with different treatments compared with control plants (infested untreated plants) during the second season (2018/2019). Bars indicate standard errors, means with the same letter over the bar are not significantly different according to Duncan’s test at 0.05 probability level. Treatment codes are as follows: M = Misr3 faba bean cultivar (tolerant to broomrape parasitism), S = Sakha3 faba bean cultivar (sensitive to broomrape parasitism C -Control (without treatments), 1-Spraying with 10% solution of SMSE, 2-Spraying + soil amendment with 10% solution of SMSE, 3-Soil amendment with 10% solution of SMSE, 4-Spraying with 10% solution of MCF, 5-Spraying + soil amendment with 10% MCF, 6-Soil amendment with 10% MCF, 7-Spraying with Roundup (144 cm^3^ ha^−1^).

Electrolyte leakage (EL) significantly increased in infested faba bean plants with broomrape (Figure 1). This increase was significant in the infested untreated plants in the sensitive cultivar (Sakha3) compared with the tolerant one (Misr3). The application of SMSE and MCF compared with the commercial herbicide Roundup (Glyphosate 48% EC) at rate 144 cm^3^ ha^-1^, caused a significant decrease in electrolyte leakage in the two cultivars especially in the tolerant one (Misr3). The seven treatments reduced electrolyte leakage in both cultivars and the best treatments were spraying with 10% solution of SMSE + soil amendment with 10% solution of SMSE (M2), and spraying with 10% solution of MCF + soil amendment with 10% MCF (M5), followed by other treatments (Figure 1). 

Broomrape parasitism also led to a significant decrease in relative water content in faba bean leaves; this decrease was greater in the sensitive cultivar (Sakha3) than in the tolerant cultivar (Misr3) (Figure 1). Additionally, our results indicate that SMSE and MCF as well as the commercial herbicide Roundup led to a significant increase in relative water content in the two cultivars, mainly in the tolerant one (Misr3). The maximum values of relative water content were recorded in the plants, which were sprayed with 10% solution of SMSE + soil amendment with 10% solution of SMSE (M2) and spraying with MCF + soil amendment with 10% MCF (M5), followed by soil amendment with 10% solution of MCF and spraying with MCF (S5).

### 2.4. Effect of Spent Mushroom Substrate Extract, Mushroom Culture Filtrate and Roundup on Leaves Anatomical Structure of Faba Bean Plants Under Broomrape Infestation

**Figure 2:**
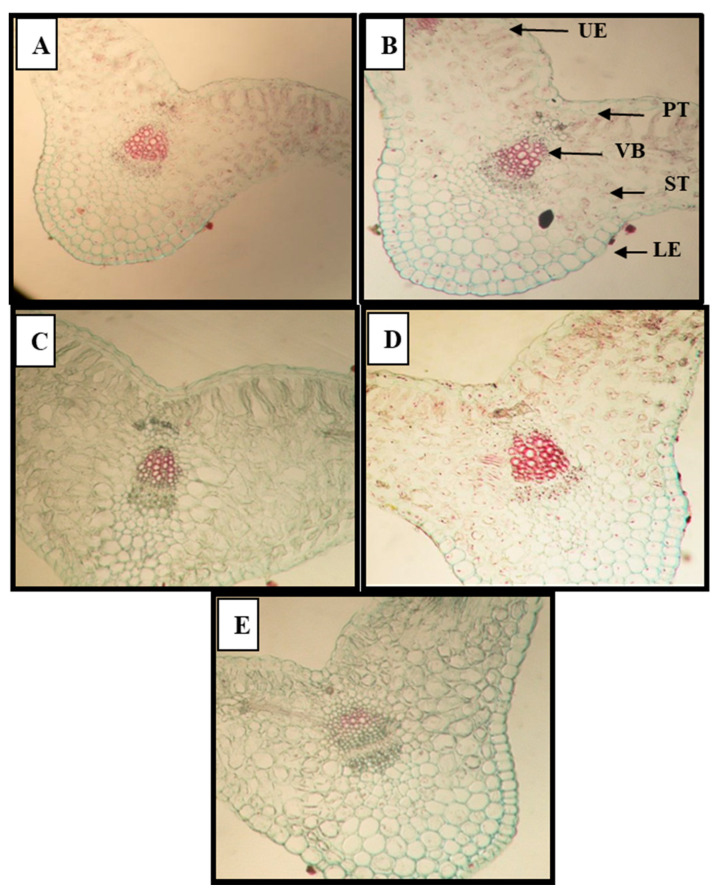
Effect of spent mushroom substrate extract (SMSE), and mushroom culture filtrate (MCF) and Roundup on anatomical characters of infested faba bean leaves (leaflet) with broomrape in the tolerant cultivar (Misr 3) during the second season (2018/2019). **A.** Control of tolerant cultivar (MC) **B**. Spraying + soil amendment with 10% solution of SMSE (M2). **C**. Spraying + soil amendment with 10% MCF (M5). **D**. Soil amendment with 10% MCF (M6). **E**. Spraying with Roundup (144 cm^3^ ha^−1^) (M7). UE—Upper epidermis. PT—Palisade tissue. ST—Spongy tissue. VB—Vascular bundles. LE—Lower epidermis.

**Figure 3:**
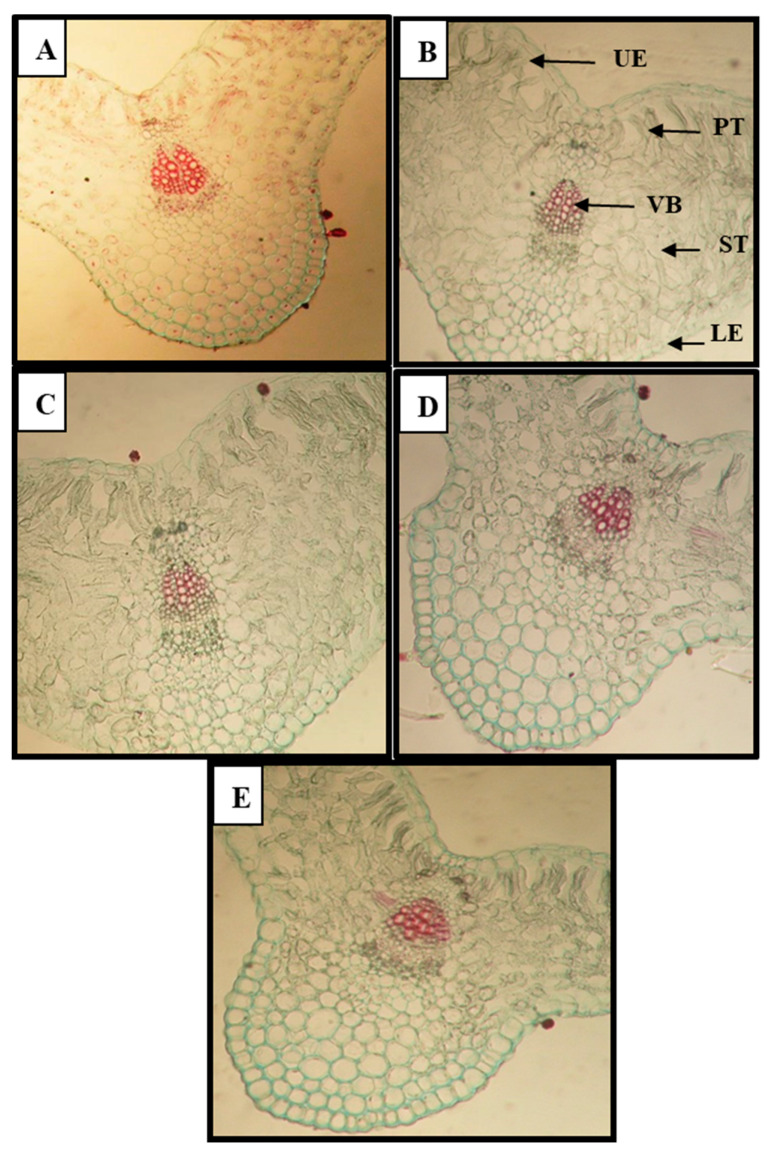
Effect of spent mushroom substrate extract (SMSE), and mushroom culture filtrate (MCF) and Roundup on anatomical characters of infested faba bean leaves (leaflet) with broomrape in the sensitive cultivar (Sakha3) during the second season (2018/2019). **A**. Control of sensitive cultivar Sakha3 (without any treatment) (SC). **B**. Spraying + soil amendment with 10% solution of SMSE (S2). **C**. Spraying + soil amendment with 10% MCF (S5). **D**. soil amendment with 10% MCF (S6). **E**. Spraying with Roundup (144 cm^3^ ha^−1^) (S7). UE—Upper epidermis. PT—Palisade tissue. ST—Spongy tissue. VB—Vascular bundles. LE—Lower epidermis.

Broomrape parasitism had adverse effects on anatomical characters of infested faba bean plants. The results obtained in Table 9 and the cross sections demonstrated in Figure 2 and Figure 3 showed that anatomical characters of faba bean leaves such as leaf lamina, palisade tissue and spongy tissue, as well as number of vessels/mid vein, were harmfully affected under broomrape infestation, compared to the infested untreated plants (control) in the two cultivars. The reduction in the studied anatomical characters of leaves in the sensitive cultivar was more than in the tolerant one (Table 9). Nevertheless, the application of SMSE and MCF as well as the commercial herbicide Roundup improved the anatomical structure of infested faba bean plants in the two cultivars, mainly in the tolerant one (Misr3), versus infested untreated plants. The best results of leaf lamina, palisade tissue and spongy tissue, number of vessels/midvein in infested treated faba bean plants were recorded with spraying with 10% solution of MCF + soil amendment with 10% MCF (M5), followed by spraying with 10% solution of SMSE + soil amendment with 10% solution of SMSE (M2), followed by the other treatments (Figure 2). However, the best results in the sensitive cultivar (Sakha3) were recorded with the S5 treatment (Figure 3).

## 3. Discussion

In the current research, ecofriendly mushroom products, spent mushroom substrate extract (SMSE) and mushroom culture filtrate (MCF) are proved to be good substitutes for the chemical herbicide Roundup. The wide use of synthetic chemicals with low specificity and low biodegradability encouraged the discovery of bio-products as templates to develop biopesticides with new chemical formulas and modes of action. Glyphosate is classified as the most widely used herbicide worldwide [10]. However, the International Agency for Research on Cancer (IARC) established in March 2015 that may cause cancer in both human and animals [22].

The application of resistant varieties only to control broomrape will reduce the diversity of faba bean strains and deprive the farmer of the advantages of other varieties, such as high productivity and quality of grains. Therefore, the induction of plant defense mechanisms through the application of products such as MCF and SMSE is a promising strategy to help the plant to complete its life cycle in the presence of pathogen without a marked reduction in yield and the application of such a strategy is reflected on whole plant health [23,24,25]. Phenolic compounds are among plant secondary metabolites that play vital roles in plant defense against biotic and a biotic stresses. Phenol,2,4-bis (1,1-dimethylethyl) and 2 (1h)-naphthalenone, octahydro-1-methyl-1-(2-p ropenyl) are the most dominant phenolic compounds in the used mushroom products SMSE and MCF, respectively. These phenolic compounds participate in the regulation of stages development, and also contribute to the defense responses during exposure to pathogen infection, extreme sunlight, heavy metal stress and injuries [26]. Such compounds often possess antioxidant activity, which is attributed to their unique chemical structure [26]. Plant defense mechanisms based on phenolic compounds include physical changes, such as increasing lignification and suberization of the plant cell walls [27], as well as metabolic changes such as the de novo synthesis of pathogenesis-related (PR) proteins [28], and biosynthesis and accumulation of phenyl propanoid secondary metabolites [29].

Besides the resistance based on physical barriers, resistance could be based on the chemical response, such as the production and secretion of the toxic phenolic compounds [30]. The sunflower–broomrape interaction is combined with increasing of phenolic level, peroxidase activity [31] and accumulation of compounds on the inner walls of host-plant xylem vessels [32,33]. Coumarins such as scopoletin, scopolin and ajapin (phenolic compounds) in sunflower inhibit the germination and attachment of broomrape [34]. Fungal metabolites were reported to affect broomrape parasitism in different plants. Aybeke [35] investigated the effects of *Fusarium oxysporum* infection on *Orobanche spp*. (broomrape) with references to change in plant hormones and secondary plant constituents. The levels of plant hormones such as indole acetic acid and gibbrilic acid in the experimental group were significantly lower than those in the control group. Moreover, infection caused the accumulation of phenolic-based compounds such as syringic acid and p-coumaric acid, which later affected broomrape development. Additionally, Tadayyon et al. [36] indicated that the application of arbuscular mycorrhizal fungi (AMF) decreased broomrape germination, the number of nodules and the dry weight of the broomrape and increased dry weight of the tomato plant as well as tomato yield under broomrape infestation.

The reduction in the growth characters of infested faba bean plants, such as plant height, root length, leaf area, and fresh and dry weights, may be due to the absorption by parasitic broomrape of key nutrients that the host plant requires for growth, such as NPK. This in turn results in many harmful effects on the host faba bean plant, such as a reduction in morphological characters and nitrogen, phosphorus and potassium contents. These results are in agreement with the results of Zayed et al. [2,4,5]. The destructive effects of broomrape on physiological characters of the faba bean plant were also recorded in our experiment. Chlorophyll a and chlorophyll b concentrations as well as relative water content were significantly reduced and electrolyte leakage was increased under broomrape parasitism. These results might be due to the negative role of broomrape on growth characters of faba bean plants and the photosynthetic process, consequently decreasing chlorophyll concentration and relative water content. These detrimental effects of broomrape (as biotic stress factor) on the physiological parameters are similar to the effects of many other stress factors on various plants: drought stress [37,38,39,40], salinity stress [41,42,43,44,45] and biotic stress factors [46,47]. Briache et al. [48] reported that Misr1 and Misr3 faba bean genotypes displayed resistance levels greater than susceptible ones; moreover, Misr1 and Misr3 gave the highest yield.

Consistent with the microscopic measurements from Figure 2 and Figure 3 and Table 10, the infested faba bean plants with broomrape showed decreased anatomical features of leaves. This reduction could be due to the deleterious effect of broomrape on growth characters and nutrient uptake. Similar results were obtained with dodder parasitism on Egyptian clover [11]. The damaging effect of stress factors on anatomical characters of plants were recorded [5,49].

The application of SMSE, MCF and Roundup improved growth and anatomical characters of faba bean plants. The use of SMSE and MCF has a qualitative advantage that distinguishes it from herbicides of chemical origin, as it works to improve vegetative characteristics of faba bean in addition to its ability to resist broomrape, in contrast to the chemical pesticide that has a bad effect on the environment, which may negatively affect the host under certain conditions.

The suppression effect of phenolic compounds such as phenolic compounds such as epicatechin, procyanidin dimer, and epigallocatechin were also observed by Moshalenko and Dementev [49] in dodder plants. Glyphosate improved growth and anatomical characters of faba bean plants because of the effective role of glyphosate in preventing the enzyme 5-enolpyruvyl-shikimate-3-phosphate synthase which reduces growth of broomrape plants [50].

Advanced separation, purification and characterization of mushroom metabolites are needed for more efficiency, in addition to search in different species of mushroom and optimization the production process through development of production media and conditions.

## 4. Materials and Methods

### 4.1. Green House Experiment

Two certified cultivars of faba bean were cultivated, one sensitive to *Orobanche crenata* (Sakha3) and the other tolerant to *Orobanche crenata* (misr 3) during the successive winter seasons in 2017/2018 and 2018/2019. The experiments were carried out in Agricultural Research Station, Sakha and in EPECRS Excellence Center, Kafrelsheikh University, Egypt. These experiments were conducted in clay soils with *Orobanche crenata* infection in pots (30 cm in diameter) with four replicates in wire greenhouses. Sowing was done on 1 November in both seasons, each pot has two seeds of faba bean. All agricultural practices for faba bean production have been done as recommended (http://www.sharkia.gov.eg/modiriat/Agriculture/Madasel.aspx?ID=15), the treatments were presented in Table 10.

### 4.2. Physical and Chemical Analysis of the Experimental Soil 

The experimental soil was assayed according to Jackson [51] and it was clay in texture. The initial soil chemical properties were: electrical conductivity (EC) 2.4, pH 8.14, organic matter (OM) 0.55%, sand 19.83, silt 31.93, clay 49.24, total nitrogen (N) 17.35 mg kg^−1^, available (P) 6.83 mg kg^−1^, and available (K) 259.36 mg kg^−1^.

### 4.3. Spent Mushroom Source and Extraction

Spent mushroom substrate extract (SMSE) of *P. ostreatus* grown on rice straw was obtained after the harvesting cycles at microbiology department, soil, water and environment research institute, Sakha Agriculture Research Station, Kafr elshikh, Egypt. The obtained spent mushroom substrate (SMS) was air dried in shade for 3 days, then dried in an oven at 70 °C until reaching a constant weight. 

### 4.4. Extraction of SMS 

Dried SMS was finely grinded and suspended in 0.5 N NaOH at a ratio (1:10) with intermittent mixing during 48 h then filtered through cheesecloth and used in subsequent work.

### 4.5. Preparation of Mushroom Culture Filtrate

Potato Dextrose broth medium was inoculated with three agar discs of 10 mm diameter obtained from the edge of a ten days old growing *P. ostreatus* on potato dextrose agar plate. The inoculated culture was incubated at 28 °C on a rotary shaker at 150 rpm for 7 days and then centrifuged at 5000 rpm for 15 min. The obtained supernatant was used in subsequent work.

### 4.6. Gas Chromatography–Mass Spectrometry (GC–MS) Analysis

The two mushroom products, SMSE and MCF, were extracted using ethyl acetate, then the chemical compositions of the samples were measured using a Trace GC-TSQ Quantum mass spectrometer (Thermo Scientific, Austin, TX, USA), with a direct capillary column TG–5MS (30 m × 0.25 mm × 0.25 µm film thickness). The column oven temperature was initially held at 50 °C and then increased by 5 °C/min to 200 °C then held for 2 min. It was then increased to the final temperature of 290 °C by 30 °C/min and held for 2 min. The injector and MS transfer line temperatures were kept at 270 and 260 °C, respectively; helium was used as a carrier gas at a constant flow rate of 1 ml/min. The solvent delay was 3 min and diluted samples of 1 µl were injected automatically using Autosampler AS1300 coupled with GC in the split mode. EI mass spectra were collected at 70 eV ionization voltages over the range of m/z 50–500 in full scan mode. The ion source temperature was set at 200 °C. The components were identified by comparison of their retention times and mass spectra with those of the WILEY 09 and NIST 11 mass spectral databases.

### 4.7. Morpho-Physiological Growth Characters of Faba Bean Plants and Broomrape Number 

Four samples were collected from each treatment then Nitrogen (N), Phosphorus (P) and Potassium (K) % were determined in plants at 60 days from sowing. Moreover, plant height (cm), root length (cm), leaf area, chlorophyll (a and b) and plant dry weight (g), number of broomrape, number of nodules/plant and nodules dry weight (g/plant) were determined at 60 days from sowing.

### 4.8. Physiological Characters

At 90 days from sowing date, the four samples of faba bean were randomly taken from each replicate to determine chlorophyll concentrations, relative water content (RWC) and electrolyte leakage (EL). The concentrations of chlorophyll a and chlorophyll b were determined according to Lichtenthaler [52] as mg g^-1^ fresh leaves using spectrophotometer at 663 and 648 nm. Electrolyte leakage was measured according to Szalai et al. [53], while relative water content was determined in fresh leaves as % according to Sanchez et al. [54] as follows:

RWC = (FW − DW)/ (TW − DW) × 100.

Fresh weight (FW) − dry weight (DW) − turgid weight (TW)

### 4.9. Morphological and Yield Characters at Harvesting Stage

At the harvesting stage, the samples of faba bean were randomly taken to determine plant height (cm), pod number/plant, seed yield g/plant, straw yield g/plant, weight of 100-seed (g).

### 4.10. Anatomical Studies

The samples of leaves (5 mm length) from the fifth leaf (terminal leaflet) were taken at the age of 60 days from the sowing date during the second season 2018/2019. The samples were killed and fixed in formalin acetic acid alcohol (FAA), washed in ethyl alcohol 50% and dehydrated in series of normal butyl alcohol according to the protocol which described by Nassar and El-Sahhar, [55]. Slides were examined and photomicrographed by light microscope (Leica Microscope Camera for Fluorescence Microscopy, Wetzlar, Germany).

### 4.11. Statistical Analysis 

The experiments were arranged in a split plot design, with four replicates. The data were statistically analyzed using the analysis of variance method as described by Gomez and Gomez [56]. The mean values of the tested treatments were compared by the least significant range (L.S.R.) according to Duncan’s Multiple Range Test [57] at *p* = 0.05 level of probability.

## 5. Conclusions

The study highlights the advantage of using eco-friendly compounds from microbial origin (i.e., mushroom (*P. ostreatus*) metabolites) to control broomrape as a substitute for commonly used herbicides, which represent environmental pollutants and can negatively affect both human and animal health. Beside their effect on the prevention of broomrape development, mushroom metabolites possessed nutritional value and their addition enhanced plant growth, chlorophyll a and chlorophyll b concentrations, relative water content and faba bean yield. Moreover, the application of mushroom metabolites improved the anatomical characters of faba bean and increased the yield component even in broomrape-tolerant varieties of faba bean.

## Figures and Tables

**Table 1:** Gas chromatography (GC) analysis of ethyl acetate extract of spent mushroom substrate extracts (SMSE).

Compound Name	Molecular Formula	Molecular Weight	CAS Number	Area
THIENO[3,4-C]PYRIDINE, 1,3,4,7-TETRAPHENYL-	C_31_H_21_NS	439	87636-67-7	3.53
1-HEXADECANOL, 2-METHYL- (Cetyl alcohol)	C_17_H_36_O	256	2490-48-4	3.96
PHENOL, 2,4-BIS(1,1-DIMETHYLETHYL)	C_14_H_22_O	206	96-76-4	58.11
1-HEXADECANOL, 2-METHYL-	C_17_H_36_O	256	2490-48-4	5.41
Cyclopentane-1-carboxylic acid, 3-acetyl-2-acetonyl-4,4-dimethyl-, methyl ester	C_14_H_22_O_4_	254	78092-00-9	0.92
1-DODECANOL, (lauryl alcohol 3,7,11-TRIMETHYL	C_15_H_32_O	228	6750-34-1	4.52
OXIRANEPENTANOIC ACID, 3-UNDECYL-, METHYL ESTER, TRANS-	C_19_H_36_O_3_	312	6175-11-7	2.36
b,4a-Epoxy-2H-cyclopenta[3,4]cyclopropa[8,9]cycloundec[1,2-b]oxiren-5(1aH)-one,2,7,9,10-tetrakis(acetyloxy)decahydro-3,6,8,8,10a-pentamethyl-	C_28_H_38_O_11_	550	51906-06-0	0.92
4,8,13-CYCLOTETRADECATRIENE-1,3-DIOL,1,5,9-TRIMETHYL-12-(1-METHYL ETHYL)-	C_20_H_34_O_2_	306	7220-78-2	0.76
1-PHENANTHRENECARBOXYLIC ACID,1,2,3,4,4A,4B,5,6,10,10A- DECAHYDRO-1,4A DIMETHYL-7-(1-METH YLETHYL)-, METHYL ESTER	C_21_H_32_O_2_	316	127-25-3	1.38
2-HEXADECANOL	C_16_H_34_O	242	14852-31-4	3.02
DOCOSANOIC ACID Behenic acid	C_23_H_46_O_5_	402	56554-25-7	0.82
PODOCARP-7-EN-3a-OL (Totarol)	C_20_H_32_O	288	4752-56-1	1.30
1,2-BENZENEDICARBOXYLIC ACID Phthalic acid	C_24_H_38_O_4_	390	117-81-7	10.91
Tetracosa-2,6,14,18,22-pentaene-10,11-diol,2,6,10,15,19,23-hexamethyl-	C_30_H_52_O_2_	444	153650-82-9	0.84
SPIROSOLAN-3-OL, 28-ACETYL-, ACETATE (ESTER)	C_31_H_49_NO_4_	499	1181-86-8	1.24

**Table 2:** GC chromatographic analyses of ethyl acetate extract of mushroom culture filtrate (MCF) of *P. ostreatus.*

Compound Name	Molecular Formula	Molecular Weight	CAS Number	Area
2-Tridecanol	C_13_H_28_O	200	1653-31-2	4.04
2(1H)-NAPHTHALENONE,OCTAHYDRO-1-METHYL-1-(2-PROPENYL)	C_14_H_22_O	206	97571-39-6	57.62
1-HEXADECANOL	C_16_H_34_O	242	36653-82-4	5.79
Propanoic acid, 2-(3-acetoxy-4,4,14-trimethylandrost-8-en-17-yl)	C_27_H_42_O_4_	430	NA	0.83
1-EICOSANOL	C_20_H_42_O	298	629-96-9	4.09
Undec-10-ynoic acid, heptadecyl ester	C_28_H_52_O_2_	420	NA	1.01
9-OCTADECENOIC ACID (Z)- 2-[(TRIMETHYLSILYL)OXY]-1-[(TRIMETHYLSILYL)OXY]METHY L]ETHYL ESTER	C_27_H_56_O_4_Si_2_	500	54284-48-9	0.71
2-PENTENOIC ACID,5-(DECAHYDRO-5,5,8A-TRIMETHYL-2- METHYLENE-1-NAPHTHALENYL)-3-METHYL-,[1S-[1à(E),4Aá,8Aà]-	C_20_H_32_O_2_	304	24470-48-2	0.82
1H-PURIN-6-AMINE, [(2-FLUOROPHENYL)METHYL]-	C_12_H_10_FN_5_	243	74421-44-6	0.67
NONACOSANOL	C_29_H_60_O	424	25154-56-7	2.72
Phthalic acid, butyl undecyl ester	C_23_H_36_O_4_	376	NA	2.36
ISOCHIAPIN B	C_19_H_22_O_6_	346	NA	0.87
4H-1-BENZOPYRAN-4-ONE,2-(3,4-DIHYDROXYPHENYL)-6,8-DI-á-D-GLUCOPYRANOSYL 5,7- DIHYDROXY-	C_27_H_30_O_16_	610	6068-80-0	0.68
2a,9a-DIHYDROXYVERRUCOSANE	C_20_H_34_O_2_	306	NA	1.37
1,2-BENZENEDICARBOXYLIC ACID	C_24_H_38_O_4_	390	117-81-7	16.43

**Table 3:** Effect of spent mushroom substrate extract (SMSE), mushroom culture filtrate (MCF), and Roundup treatments to control broomrape infestation, on percent (%) content of nitrogen (N), potassium (K) and phosphorous (P) in faba bean plants at 60 days from sowing, during two seasons.

Treatments ^1^	N % in Plant		K % in Plant		P % in Plant	
	1st Season	2nd Season	1st Season	2nd Season	1st Season	2nd Season
**MC**	1.13	1.05	1.16	1.18	0.21	0.20
**M1**	1.36	1.34	1.26	1.27	0.25	0.25
**M2**	1.42	1.42	1.36	1.34	0.31	0.31
**M3**	1.23	1.21	1.27	1.25	0.26	0.25
**M4**	1.65	1.58	1.44	1.40	0.31	0.29
**M5**	1.70	1.72	1.72	1.67	0.32	0.28
**M6**	1.51	1.52	1.54	1.49	0.26	0.26
**M7**	1.33	1.31	1.28	1.27	0.24	0.24
**Sc**	1.09	1.06	1.08	1.05	0.20	0.19
**S1**	1.31	1.29	1.19	1.16	0.24	0.23
**S2**	1.35	1.37	1.25	1.22	0.31	0.31
**S3**	1.22	1.22	1.19	1.23	0.26	0.24
**S4**	1.53	1.50	1.31	1.34	0.30	0.30
**S5**	1.63	1.62	1.42	1.41	0.31	0.31
**S6**	1.43	1.41	1.37	1.34	0.27	0.26
**S7**	1.32	1.30	1.16	1.17	0.23	0.22
**L.S.D ^2^. 0.05**	0.036	0.062 **	0.086 **	0.059 **	0.029 **	0.034 **

^1^ Treatment codes are as follows: M = Misr3 faba bean cultivar (tolerant to broomrape parasitism), S = Sakha3 faba bean cultivar (sensitive to broomrape parasitism C -Control (without treatments), 1-Spraying with 10% solution of SMSE, 2-Spraying + soil amendment with 10% solution of SMSE, 3-Soil amendment with 10% solution of SMSE, 4-Spraying with 10% solution of MCF, 5-Spraying + soil amendment with 10% MCF, 6-Soil amendment with 10% MCF, 7-Spraying with Roundup (144 cm^3^ ha^−1^); ^2^ Least Significant Difference, determined at 5% probability. The presence of asterisks indicates that there were statistically significant differences between treatments. ** *p* < 0.01.

**Table 4:** Effect of spent mushroom substrate extract (SMSE), mushroom culture filtrate (MCF), and Roundup® treatments to control broomrape infestation on plant dry weight (g), plant height and root length in faba bean plants at 60 days from sowing during two seasons.

Treatments ^1^	Plant Dry Weight (g)		Plant Height (cm)		Root Length (cm)	
	1st Season	2nd Season	1st Season	2nd Season	1st Season	2nd Season
**MC**	14.33	14.57	66.00	66.00	23.00	23.00
**M1**	15.25	17.33	66.00	65.33	37.00	35.00
**M2**	17.20	16.93	69.00	66.00	33.00	31.00
**M3**	16.07	16.27	80.00	80.67	33.00	34.33
**M4**	21.07	21.27	74.00	76.00	31.00	32.00
**M5**	22.63	22.93	82.67	85.00	39.33	40.00
**M6**	19.20	19.52	88.33	87.67	36.67	37.33
**M7**	15.70	15.27	77.00	77.00	27.67	28.00
**SC**	14.22	14.23	64.33	70.67	24.00	22.67
**S1**	17.35	17.13	71.33	77.33	35.00	34.33
**S2**	18.77	19.40	78.33	80.33	34.33	34.67
**S3**	19.13	19.50	81.00	84.33	32.00	32.00
**S4**	17.43	17.03	81.33	79.33	31.33	34.33
**S5**	20.00	19.28	89.67	87.67	40.00	37.00
**S6**	17.63	17.23	81.33	83.33	38.00	38.00
**S7**	15.33	15.07	70.67	74.67	20.00	21.33
**L.S.D ^2^. 0.05**	3.13 **	3.01 **	4.36 **	5.17 **	3.73 **	2.92 **

^1^ Treatment codes are as follows: M = Misr3 faba bean cultivar (tolerant to broomrape parasitism), S = Sakha3 faba bean cultivar (sensitive to broomrape parasitism C -Control (without treatments), 1-Spraying with 10% solution of SMSE, 2-Spraying + soil amendment with 10% solution of SMSE, 3-Soil amendment with 10% solution of SMSE, 4-Spraying with 10% solution of MCF, 5-Spraying + soil amendment with 10% MCF, 6-Soil amendment with 10% MCF, 7-Spraying with Roundup (144 cm^3^ ha^−1^); ^2^ Least Significant Difference, determined at 5% probability. The presence of asterisks indicates that there were statistically significant differences between treatments. ** *p* < 0.01.

**Table 5:** Effect of spent mushroom substrate extract (SMSE), mushroom culture filtrate (MCF), and Roundup® treatments to control broomrape infestation on leaf area (cm^2^), chlorophyll a and chlorophyll b of faba bean plants at 60 days from sowing during two seasons.

Treatments ^1^	Leaf Area (cm^2^)		Chlorophyll a		Chlorophyll b	
	1st Season	2nd Season	1st Season	2nd Season	1st Season	2nd Season
**MC**	62.72	63.27	13.43	13.48	10.52	9.96
**M1**	74.34	72.65	14.16	14.01	12.25	12.47
**M2**	106.31	105.81	15.00	14.93	13.09	12.59
**M3**	130.73	135.26	14.36	14.34	12.53	12.75
**M4**	110.16	107.99	16.97	16.84	13.59	13.70
**M5**	141.24	138.54	25.80	17.14	20.14	19.07
**M6**	135.77	137.45	16.21	16.28	14.04	14.43
**M7**	129.81	123.26	10.82	10.80	7.57	7.44
**SC**	60.54	60.98	12.55	12.57	11.43	10.80
**S1**	76.58	81.59	16.03	16.23	11.56	11.30
**S2**	91.56	93.92	16.34	16.49	12.47	12.36
**S3**	111.74	111.27	16.02	15.99	13.91	13.65
**S4**	95.20	93.81	20.05	19.53	15.29	15.16
**S5**	119.05	112.36	22.42	22.30	16.46	16.00
**S6**	74.65	61.63	19.78	19.47	15.78	15.44
**S7**	72.01	66.54	9.50	9.29	6.71	6.66
**L.S.D ^2^. 0.05**	14.64 **	14.80 **	0.51 **	0.67 **	0.58 **	1.06 **

^1^ Treatment codes are as follows: M = Misr3 faba bean cultivar (tolerant to broomrape parasitism), S = Sakha3 faba bean cultivar (sensitive to broomrape parasitism C -Control (without treatments), 1-Spraying with 10% solution of SMSE, 2-Spraying + soil amendment with 10% solution of SMSE, 3-Soil amendment with 10% solution of SMSE, 4-Spraying with 10% solution of MCF, 5-Spraying + soil amendment with 10% MCF, 6-Soil amendment with 10% MCF, 7-Spraying with Roundup (144 cm^3^ ha^−1^); ^2^ Least Significant Difference, determined at 5% probability. The presence of asterisks indicates that there were statistically significant differences between treatments. ** *p* < 0.01.

**Table 6:** Effect of spent mushroom substrate extract (SMSE), mushroom culture filtrate (MCF), and Roundup® treatments to control broomrape infestation on broomrape number (No.), No. of Rhizobia nodules/plant, and Nodules dry weight (g/plant) at 60 days from sowing.

Treatments ^1^	No. Broomrape		No. Nodules/Plant		Nodules Dry we./Plant (g)	
	1st Season	2nd Season	1st Season	2nd Season	1st Season	2nd Season
**MC**	1.33	1.67	21.00	19.00	0.16	0.15
**M1**	0.67	0.33	64.33	63.00	0.21	0.22
**M2**	0.33	0.33	75.67	72.33	0.27	0.25
**M3**	0.67	0.67	42.33	37.33	0.31	0.31
**M4**	0.33	0.67	70.33	72.67	0.31	0.31
**M5**	0.00	0.00	71.67	68.33	0.37	0.39
**M6**	0.00	0.00	45.67	53.00	0.28	0.29
**M7**	0.33	0.67	20.00	21.33	0.16	0.14
**SC**	3.33	3.67	40.67	39.67	0.14	0.14
**S1**	3.67	3.33	56.67	59.67	0.23	0.24
**S2**	2.00	2.33	50.00	53.33	0.24	0.26
**S3**	3.33	3.67	46.67	48.33	0.23	0.23
**S4**	2.33	2.67	44.33	49.67	0.23	0.26
**S5**	1.33	1.67	68.00	66.67	0.28	0.29
**S6**	1.67	1.33	43.67	43.67	0.20	0.20
**S7**	1.00	0.67	20.33	24.33	0.15	0.15
**L.S.D ^2^. 0.05**	0.93 **	0.98 **	8.36 **	6.54 **	0.06 **	0.041 **

^1^ Treatment codes are as follows: M = Misr3 faba bean cultivar (tolerant to broomrape parasitism), S = Sakha3 faba bean cultivar (sensitive to broomrape parasitism C -Control (without treatments), 1-Spraying with 10% solution of SMSE, 2-Spraying + soil amendment with 10% solution of SMSE, 3-Soil amendment with 10% solution of SMSE, 4-Spraying with 10% solution of MCF, 5-Spraying + soil amendment with 10% MCF, 6-Soil amendment with 10% MCF, 7-Spraying with Roundup (144 cm^3^ ha^−1^); ^2^ Least Significant Difference, determined at 5% probability. The presence of asterisks indicates that there were statistically significant differences between treatments. ** *p* < 0.01.

**Table 7:** Effect of spent mushroom substrate extract (SMSE), mushroom culture filtrate (MCF), and Roundup® treatments to control broomrape infestation on plant dry weight, plant height and pod numbers of faba bean plants at harvesting stage during two seasons.

Treatments ^1^	Dry Weight of Plant (g)		Plant Height (cm)		Pod Number/Plant	
	1st Season	2nd Season	1st Season	2nd Season	1st Season	2nd Season
**MC**	15.98	15.23	84.77	77.30	1.67	1.67
**M1**	23.03	22.39	88.88	88.38	3.33	3.67
**M2**	26.57	25.99	107.37	104.70	5.67	5.33
**M3**	25.73	25.49	104.83	104.07	5.33	5.33
**M4**	24.35	23.79	107.03	105.70	6.00	6.33
**M5**	35.94	35.31	119.57	117.60	8.67	8.00
**M6**	30.37	30.05	107.67	103.60	8.33	8.00
**M7**	21.03	20.17	85.10	85.03	2.33	2.00
**SC**	14.20	14.37	82.37	83.14	1.33	1.00
**S1**	22.48	22.04	94.33	89.57	3.33	3.33
**S2**	26.09	25.34	103.67	102.23	6.00	5.33
**S3**	25.32	24.52	99.93	91.97	5.00	5.00
**S4**	25.05	23.97	101.93	92.07	5.67	6.00
**S5**	34.97	34.21	117.43	113.77	7.67	7.33
**S6**	28.03	27.49	109.00	105.90	7.00	6.67
**S7**	17.07	16.57	80.70	74.07	1.67	1.33
**L.S.D ^2^. 0.05**	2.04 **	2.10 **	6.61 **	9.69 **	1.44 **	1.27 **

^1^ Treatment codes are as follows: M = Misr3 faba bean cultivar (tolerant to broomrape parasitism), S = Sakha3 faba bean cultivar (sensitive to broomrape parasitism C -Control (without treatments), 1-Spraying with 10% solution of SMSE, 2-Spraying + soil amendment with 10% solution of SMSE, 3-Soil amendment with 10% solution of SMSE, 4-Spraying with 10% solution of MCF, 5-Spraying + soil amendment with 10% MCF, 6-Soil amendment with 10% MCF, 7-Spraying with Roundup (144 cm^3^ ha^−1^); ^2^ Least Significant Difference, determined at 5% probability. The presence of asterisks indicates that there were statistically significant differences between treatments. ** *p* < 0.01.

**Table 8:** Effect of spent mushroom substrate extract (SMSE), mushroom culture filtrate (MCF), and Roundup® treatments to control broomrape infestation on faba bean yield parameters at harvesting stage during two seasons.

Treatments ^1^	Seed Yield (g/Plant)		Straw Yield (g/Plant)		100-Seed (g)	
	1st Season	2nd Season	1st Season	2nd Season	1st Season	2nd Season
**MC**	6.86	6.64	7.34	7.17	39.53	40.64
**M1**	8.93	8.71	8.67	8.92	42.05	41.34
**M2**	9.24	8.83	10.11	10.51	52.98	52.44
**M3**	8.35	8.11	9.37	9.50	48.42	47.59
**M4**	9.87	9.45	11.05	11.32	47.66	45.87
**M5**	13.00	12.72	15.42	15.14	61.47	60.47
**M6**	12.23	11.69	12.15	12.42	57.78	56.76
**M7**	8.72	8.48	8.63	8.69	40.36	39.76
**Sc**	5.51	5.89	6.14	6.02	38.75	37.32
**S1**	6.47	6.28	7.60	7.52	41.87	41.02
**S2**	7.76	7.91	8.54	8.80	52.13	51.48
**S3**	7.64	7.50	7.94	7.65	47.42	46.73
**S4**	8.36	7.91	9.64	10.03	45.37	44.70
**S5**	10.00	9.86	10.62	10.33	59.43	58.77
**S6**	9.34	9.01	10.36	10.62	56.18	55.47
**S7**	6.15	6.17	7.83	7.85	39.12	38.48
**L.S.D ^2^. 0.05**	1.04 **	0.95 **	0.89 **	0.99 **	2.42 **	1.87 **

^1^ Treatment codes are as follows: M = Misr3 faba bean cultivar (tolerant to broomrape parasitism), S = Sakha3 faba bean cultivar (sensitive to broomrape parasitism C -Control (without treatments), 1-Spraying with 10% solution of SMSE, 2-Spraying + soil amendment with 10% solution of SMSE, 3-Soil amendment with 10% solution of SMSE, 4-Spraying with 10% solution of MCF, 5-Spraying + soil amendment with 10% MCF, 6-Soil amendment with 10% MCF, 7-Spraying with Roundup (144 cm^3^ ha^−1^); ^2^ Least Significant Difference, determined at 5% probability. The presence of asterisks indicates that there were statistically significant differences between treatments. ** *p* < 0.01.

**Table 9:** Effect of spent mushroom substrate extract (SMSE), mushroom culture filtrate (MCF), and Roundup® treatments to control broomrape infestation on anatomical characters of infested faba bean leaves (leaflet) with broomrape.

Treatments	Thickness of Lamina (µm)	Thickness of Spongy Tissue (µm)	Thickness of Palisade Tissue (µm)	Number of Vessels/Midvein
**MC**	353.35	221.45	87.56	27.6
**M1**	362.24	227.67	91.42	31.5
**M2**	396.04	238.95	103.19	35
**M3**	375.05	221.72	96.15	30.4
**M4**	381.72	224.45	98.23	33
**M5**	403.42	243.02	106.28	34.5
**M6**	374.76	222.83	93.54	32.3
**M7**	378.32	230.44	97.35	33.2
**Sc**	349.91	221.66	89.54	25.7
**S1**	357.78	226.18	95.75	28.5
**S2**	387.51	235.05	99.92	32.1
**S3**	368.65	226.70	92.66	30.2
**S4**	372.29	231.09	94.14	31.5
**S5**	389.98	238.14	97.05	32.9
**S6**	364.23	229.18	89.23	31.2
**S7**	354.54	225.42	87.57	31.7

Treatment codes are as follows: M = Misr3 faba bean cultivar (tolerant to broomrape parasitism), S = Sakha3 faba bean cultivar (sensitive to broomrape parasitism C -Control (without treatments), 1-Spraying with 10% solution of SMSE, 2-Spraying + soil amendment with 10% solution of SMSE, 3-Soil amendment with 10% solution of SMSE, 4-Spraying with 10% solution of MCF, 5-Spraying + soil amendment with 10% MCF, 6-Soil amendment with 10% MCF, 7-Spraying with Roundup (144 cm^3^ ha^−1^).

**Table 10:** Treatments of greenhouse experiments during the two seasons.

Treatments	
**Mc**	Control of tolerant cultivar Misr3 (without any treatment)
M1	Spraying with 10% solution of spent mushroom substrate extract ( Misr3)
M2	Spraying + soil amendment with 10% solution of spent mushroom substrate extract (Misr3)
M3	soil amendment with 10% solution of spent mushroom substrate extract (Misr3 )
M4	Spraying with 10% solution culture filtrate (Misr3 )
M5	Spraying + soil amendment with 10% mushroom culture filtrate (Misr3)
M6	soil amendment with 10% mushroom culture filtrate (Misr3)
M7	Spraying with Roundup (144 cm^3^ ha^−1^) (Misr3)
Sc	Control of sensitive cultivar Sakha3 (without any treatment)
S1	Spraying with 10% solution of spent mushroom substrate extract (Sakha3)
S2	Spraying + soil amendment with 10% solution of spent mushroom substrate extract (Sakha3)
S3	soil amendment with 10% solution of spent mushroom substrate extract (Sakha3)
S4	Spraying with 10% solution of mushroom culture filtrate (Sakha3)
S5	Spraying + soil amendment with 10% mushroom culture filtrate (Sakha3)
S6	soil amendment with 10% mushroom culture filtrate (Sakha3)
S7	Spraying with Roundup (144 cm^3^ ha^−1^) (Sakha3)
